# Supplementary material for: TGF-β-Induced PAUF Plays a Pivotal Role in the Migration and Invasion of Human Pancreatic Ductal Adenocarcinoma Cell Line Panc-1
Source: Int J Mol Sci. 2024 Oct 24;25(21):11420. doi: 10.3390/ijms252111420 (PMC11546992; doi:10.3390/ijms252111420)
Supplement: Supplementary file 1 [file ijms-25-11420-s001.zip › ijms-3282195-supplementary.pdf]

TGF-β-induced PAUF Plays a Pivotal Role in Migration and Invasion of Human Pancreatic Ductal Adenocarcinoma Cell Line Panc-1

Supplementary materials

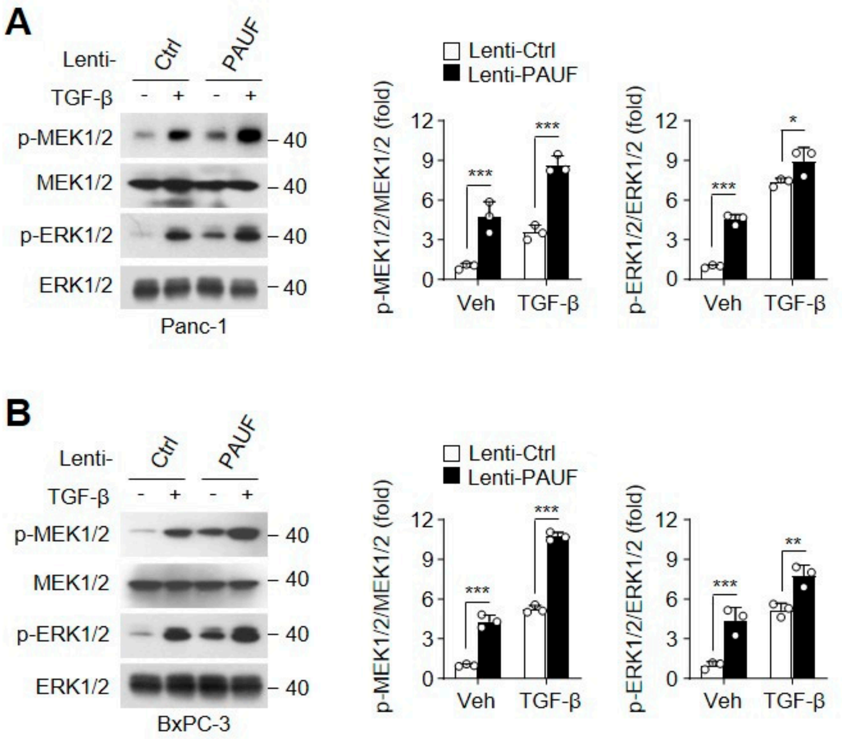

Supplementary Figure S1. Overexpression of pancreatic adenocarcinoma upregulated factor (PAUF) augments TGF-β-mediated MEK1/2 and ERK1/2 activation. **(A)** Panc-1 cells were transfected with Lenti-Ctrl or Lenti-PAUF expression vectors and treated with vehicle or TGF-β (10 ng/mL) for 1 h. Phosphorylated MEK1/2 and ERK1/2 levels were determined using immunoblotting, and densitometric values of phospho-MEK1/2 and -ERK1/2 were normalized against total MEK1/2 and ERK1/2 ($n = 3$). **(B)** Lenti-Ctrl- or Lenti-PAUF-transfected BxPC-3 cells were stimulated with vehicle or TGF-β (10 ng/mL) for 1 h. Analysis of MEK1/2 and ERK1/2 phosphorylation levels was performed as described in **(A)**. Statistical significance was calculated using two-way ANOVA followed by *post-hoc* multiple comparisons test with Bonferroni correction. Data are presented as the mean ± standard deviation (SD). * $p < 0.05$, ** $p < 0.01$, *** $p < 0.001$.
